# Supplementary material for: Computational modeling of multiscale collateral blood supply in a whole-brain-scale arterial network
Source: PLoS Comput Biol. 2023 Sep 8;19(9):e1011452. doi: 10.1371/journal.pcbi.1011452 (PMC10519592; doi:10.1371/journal.pcbi.1011452)
Supplement: Attachment — Submitted filename: Review for the article.pdf [file pcbi.1011452.s001.pdf]

Review for the article titled:
"Computational modeling of multiscale collateral blood supply in a whole-brain-scale arterial network"

The authors develop a computational model based on a large scale 1D cerebral arterial networks (generated in their previous paper) and on zero dimensional blood flow equations. This computational approach is very interesting and is used in this paper to help understand the impact of anastomoses on the supply of blood in certain physiological conditions such as occlusions. The authors include several numerical computations which in normal conditions agree with measured data.

Please find below my comments which I think constitute a major revision to the article. I recommend it for publication after the points below are sufficiently addressed.

- In the methods section, does " Ω_{i}^c" define a region specified from one surface artery ? I suggest including a more precise definition of this and of "N_1" as it is not immediately clear from the text.
- The authors refer to their previous article for the tree structure they use here. Looking at the data provided in this article, as seen in Figure 1a, the surface arteries seem to intersect the brain hemispheres. In their previous article, the authors mention that this was corrected where the vessels were adjusted to the brain surface (boundary). Is the corrected network being used in the present analysis ? If yes, I suggest sharing the corrected network data in the zenodo link. If not, I suggest including a remark in the present paper explaining this and how it may be a limitation of the present study.
- How does the value of "q_i^c" defined in the "whole brain scale arterial network model" section relate to the equations described in the blood flow simulations section? Does it constitute a prescribed boundary condition ? or is it determined from the solution of the blood flow equations? I think this should be made clear.
- In the blood flow simulation section, equation (2), the notation should be " $\partial_{ss}^2 p = 0$" instead of " $\partial_{s}^2 p = 0$".
- I suggest that the authors comment on the conditions or assumptions enforced at the bifurcation points of the arterial tree. Are conservation of mass and continuity of fluxes being enforced ?
- I suggest the authors comment on the limitations of the Poiseuille flow assumption and what it entails (fixed radius, …) It will also be a good idea to give a reference for the derivation of Poiseuille flow where the required assumptions are clearly stated.
- I suggest the authors mention the type of the Galerkin finite element method used (continuous Galekrin? , polynomial degree, mesh size).
- I suggest that the authors include the definitions of N_1 and N_2 again in line 195 to aid the reader.

- Include a clear definition of “N” in line 209. While it was previously stated “N numbers potential candidates as intermediate anastomoses”, it is still unclear to me whether N enumerates the vessels that can be connected ? or the possible pairs ? Namely it is still unclear to me how we have 2^N possible combinations in line 209 which suggests that we have N different connections.
- In the paragraph starting on line 229, I suggest that the authors comment on why they chose these particular values for N_1 and N_2 as a representative case ? why is this a good representative case ?
- In the paragraph starting on line 229, is the flow recovery ratio the same as the one described in lines 138-140 ? If so, the authors should refer to it for readability.
- Linking to the above comment, the authors show “spatial distributions of the flow recovery ratio”. How is this being computed ? if the same process as the one described in 138-140 is used, then this is a constant quantity in each subregion. I recommend including a more precise description.
- In Figure 3, include one or two sentences describing the observations from the plot to help the reader.
- In Figure 5, it shows a spatial distribution of flow on the surface of the brain. I think that a precise description of the way this is being computed should be included. The process mentioned in line 138-140 can be made more precise and can be referred to throughout.
