# Supplementary material for: Computational modeling of multiscale collateral blood supply in a whole-brain-scale arterial network
Source: PLoS Comput Biol. 2023 Sep 8;19(9):e1011452. doi: 10.1371/journal.pcbi.1011452 (PMC10519592; doi:10.1371/journal.pcbi.1011452)
Supplement: Attachment — Submitted filename: Response_letter_v3.docx [file pcbi.1011452.s002.docx]

**Response Letter**

We thank the editor and the reviewers for taking the time to review our manuscript and provide valuable comments and suggestions to further improve it. Listed below are point-by-point responses to all the comments from the reviewers. We have also made a revision of the manuscript according to our responses, where major changes are highlighted in yellow in the revised manuscript.

**Reviewer #1**:

**Comment 1. In the methods section, does “ \Omega_{i}^c” define a region specified from one surface artery ? I suggest including a more precise definition of this and of “N_1” as it is not immediately clear from the text.**

*Response*

Yes,$\Omega_{i}^{c}$(i=1,…, *N*_1_) define vascular subregions specified from each terminal of the coarse arteries, where *N*_1_ is the number of coarse arterial subregions. In the revised manuscript, we have clarified the definition of$\Omega_{i}^{c}$ and *N*_1_ more clearly on page 6, as follows.

“Each terminal of the coarse arteries is assigned at each subregion $\Omega_{i}^{c}$($i=1,\ldots,N_{1}$) of the brain cortical surface, where *N*_1_ is the number of coarse arterial subregions.”

**Comment 2. The authors refer to their previous article for the tree structure they use here. Looking at the data provided in this article, as seen in Figure 1a, the surface arteries seem to intersect the brain hemispheres. In their previous article, the authors mention that this was corrected where the vessels were adjusted to the brain surface (boundary). Is the corrected network being used in the present analysis ? If yes, I suggest sharing the corrected network data in the zenodo link. If not, I suggest including a remark in the present paper explaining this and how it may be a limitation of the present study.**

*Response*

As the reviewer pointed out, the surface arteries reconstructed from medical images partially intersect the brain hemispheres because the geometries of the brain hemispheres and the surface arteries were extracted from different subjects due to difficulties of data accessibility (Ii et al. 2020). In the previous study, we manually adjusted the surface arteries to fit the brain hemispheres by affine transformation (the adjusted arterial geometry was posted in the Zendo link shown in the previous study), while local intersections between these geometries remained. These intersections do not affect the subsequent modeling of the arterial network because this process uses only the terminals of the large arteries, while these inconsistencies lose the information regarding personal specificity, as noted in the limitations section of our previous paper (Ii et al. 2020). We have added the above point in the limitations section on pages 14-15, as follows.

“For example, the geometries of the brain surface and the large artery reconstructed from medical images used in this study were extracted from different subjects due to difficulties of data accessibility (21). Although this inconsistency does not affect the subsequent modeling of the arterial network because only the terminal nodes of the large arteries were used in this process, this limitation loses information regarding personal specificity, as noted in (21).”

**Comment 3. How does the value of “q_i^c” defined in the “whole brain scale arterial network model” section relate to the equations described in the blood flow simulations section? Does it constitute a prescribed boundary condition ? or is it determined from the solution of the blood flow equations? I think this should be made clear.**

*Response*

The value of $q_{i}^{c}$ is the flow rate in each coarse artery and is used in the arterial network modeling only to represent the assumption of equal flow rate in the brain cortex, and is not related to the equations in the blood flow simulation. This study considers multiscale anastomoses and middle cerebral artery occlusion, and thus the blood flow distribution in the coarse arteries is not prescribed but determined by the blood flow simulations. To avoid this confusion, we have added the above point on page 7 in the revised manuscript, as follows.

“Note that the flow rate $q_{i}^{c}$ was only used in the process of the coarse arterial network modeling process and was not applied as a prescribed boundary condition in the subsequent blood flow simulation.”

**Comment 4. In the blood flow simulation section, equation (2), the notation should be “ \partial_{ss}^2 p = 0” instead of “ \partial_{s}^2 p = 0”.**

*Response*

As noted in the response to comment 5, we have changed equation (2) from the continuous form to the discrete form for readability.

**Comment 5. I suggest that the authors comment on the conditions or assumptions enforced at the bifurcation points of the arterial tree. Are conservation of mass and continuity of fluxes being enforced ?**

*Response*

As commented, we impose the mass conservation (flow rate conservation) and pressure continuity at the bifurcation points of the arterial tree. The weak form of the governing equation $\partial_{s}\left(\frac{\pi d^{4}}{128\mu}\partial_{s}p\right)=0$ $\left(s\in\left[0,L\right]\right)$, which comes from the mass conservation equation$\left(\partial_{s}q=0\right)$ combined with the equation of motion　$\left(\partial_{s}p=\frac{128\mu}{\pi d^{4}}q\right)$, is written by

$-\int_{0}^{L} \left(\frac{\pi d^{4}}{128\mu}\partial_{s}\hat{p}\partial_{s}p\right)ds+\left(\left.\hat{p}\right|_{L}-\left.\hat{p}\right|_{0}\right)q=0$,	(A1)

where$\hat{p}$ is the test function. In our study, we assume the diameter in each edge is constant, i.e.,$d\left(s\right)\equiv d$, and thus the linear polynomial gives the exact distribution for *p* because$\partial_{ss}^{2}p=0$ holds in each edge. When an edge is treated as a two-node linear line element *ij* with nodes *i* and *j*, the resulting algebraic form of the eq. (A1) is given by

$k_{ij}\left(\begin{matrix} 1 & -1 \\ -1 & 1 \end{matrix}\right)\left(\begin{matrix} p_{i} \\ p_{j} \end{matrix}\right)=\left(\begin{matrix} -1 \\ 1 \end{matrix}\right)q_{ij},k_{ij}=\frac{\pi d_{ij}^{4}}{128\mu L_{ij}}$.	(A2)

By assembling eq. (A2) of all edges in a whole arterial network using the pressure continuity and mass conservation at the bifurcation points, the mass balance equation of the node *i* can be derived as

$\sum_{j}^{n_{i}} k_{ij}\left(p_{i}-p_{j}\right)=s_{i}$,	(A3)

where *n_i_* is the number of connecting nodes to node *i* and *s_i_* is the source term and is set to zero except for the inlet and outlet boundaries.

As noted, the finite element framework has an advantage for numerical accuracy with considering the spatial distribution of the diameter; however, under the condition with a constant diameter in each edge, the above formulation is equivalent to the following zero-dimensional discrete expression:

$q_{ij}=-k_{ij}\left(p_{i}-p_{j}\right)$.	(A4)

Therefore, we have changed the description of the formulation to the discrete form for readabilities, on page 8 in the revised manuscript, as follows.

“Poiseuille flow was assumed in each artery; thus, the flow rate *q_ij_* in an edge *ij* with the node *i* and *j* was given by

$q_{ij}=-k_{ij}\left(p_{i}-p_{j}\right),\text{ }k_{ij}=\frac{\pi d_{ij}^{4}}{128\mu L_{ij}}$,

where *p_i_* and *p_j_* are the pressure at the node *i* and *j*, *d_ij_* is the edge diameter, *L_ij_* is the edge length, and *k_ij_* is the constant determined from the Poiseuille flow assumption. To satisfy the mass conservation at the branching point, the mass-balance equation at the node *i* was given by

$$\sum_{j}^{n_{i}} k_{ij}\left(p_{i}-p_{j}\right)=s_{i}$$

Where *n_i_* is the number of nodes connecting to node *i*, and *s_i_* is the source term and is set to zero except for the boundaries. The linear algebraic equation about the pressure was constructed by assembling eq. (2) of all nodes in a whole arterial network.”

**Comment 6. I suggest the authors comment on the limitations of the Poiseuille flow assumption and what it entails (fixed radius, … ) It will also be a good idea to give a reference for the derivation of Poiseuille flow where the required assumptions are clearly stated.**

*Response*

Because the velocity profile varies throughout the arterial system, from being almost flat in the aorta to a more parabolic shape in the peripheral arteries (Olufsen 1999), we assumed that the velocity profile in the cerebral arterial network, which has a relatively small diameter compared to the aorta, is the Poiseuille flow with a parabolic shape. However, this assumption has several limitations, as noted by the reviewer. In cerebral arterial networks, flow unsteadiness with arterial wall deformation occurs in large arteries around the circle of Willis, whereas non-Newtonian flow characteristics may occur in the small peripheral arteries. We believe that the Poiseuille flow assumption is reasonable to clarify the qualitative flow distributions in an entire arterial network, further consideration of the above multiscale flow profiles would provide a detailed quantitative understanding of the cerebral blood supply. In the revised manuscript, we have added the above limitation on page 15, as follows.

“Fourth, the arterial modeling and blood flow simulation has several assumptions. Because the flow profiles in the peripheral arteries are parabolic shape rather than flat as observed in the aorta (41), we assumed the flow profiles in the whole cerebral arteries to be the steady Poiseuille flow. However, flow unsteadiness and associated arterial deformations may occur in large arteries around the circle of Willis, while non-Newtonian properties may not be negligible in the small arteries. Further consideration of the above multiscale flow profiles in a whole cerebral arterial structure would provide a detailed quantitative understanding of the cerebral hemodynamics.”

**Comment 7. I suggest the authors mention the type of the Galerkin finite element method used (continuous Galekrin? , polynomial degree, mesh size ).**

*Response*

As noted in the response to comment 5, we used the continuous Galerkin finite element method to derive the mass balance equation. Each edge was treated as a two-node linear line element, and thus the element (mesh) size is equal to the edge length. In the revised manuscript, we have changed the description of the formulation to the discrete form for readability.

**Comment 8. I suggest that the authors include the definitions of N_1 and N_2 again in line 195 to aid the reader.**

*Response*

Following the reviewer’s suggestion, we have added the definition of *N*_1_ and *N*_2_ on page 9 in the revised manuscript, as follows.

“we set three patterns of *N*_1_ and *N*_2_ (the number of coarse and fine arterial subregions)”

**Comment 9. Include a clear definition of “N” in line 209. While it was previously stated “N numbers potential candidates as intermediate anastomoses”, it is still unclear to me whether N enumerates the vessels that can be connected ? or the possible pairs ? Namely it is still unclear to me how we have 2^N possible combinations in line 209 which suggests that we have N different connections.**

*Response*

In this study, we considered intermediate anastomoses at boundaries between territories of RMCA and adjacent arteries (RACA and RPCA), and all terminal pairs of nearest neighbors between these territories were selected as potential candidates to assign the intermediate anastomoses (*N*: total number of the potential candidates). Because there are two ways to assign the anastomosis or not at each pair, the total number of combinations of assigning the intermediate anastomoses from *N* pairs is 2*^N^*.

In the revised manuscript, we have brushed up the description of the definition of *N* and the number of combinations on pages 7 and 10, as follows.

On page 7:

“In the construction of intermediate anastomoses, boundaries between territories of the RMCA and adjacent main cerebral artery (RACA and RPCA) were segmented by coarse arterial terminals, and all terminal pairs of nearest neighbors between these territories (total number of *N*) were then selected as potential candidates to assign the intermediate anastomoses (Fig. 2(b)). Because of the sparse properties of intermediate anastomoses (12), we adopted *n* numbers of pairs from *N* numbers of potential candidates to assign intermediate anastomoses.”

On page 10:

“Because there are two ways to assign the anastomosis or not at each terminal pair selected as a potential candidate (Fig. 2(b)), the total number of combinations of the assignment of the intermediate anastomoses from *N* pairs are very large (2*^N^*).”

**Comment 10. In the paragraph starting on line 229, I suggest that the authors comment on why they chose these particular values for N_1 and N_2 as a representative case ? why is this a good representative case ?**

*Response*

Effects of *N*_1_ and *N*_2_ on the vascular morphology were evaluated in our previous study and we concluded that these effects were secondary at least in cases of *N*_1_ =1000, 2000, and 4000 (Ii et al., 2020). However, smaller *N*_1_ leads to larger terminal diameters of the coarse arterial networks (Fig. 3) compared to those of actual intermediate anastomoses (200-610 μm (Vander Eecken and Adams 1953)). Therefore, we chose *N*_1_ = 2000 as a representative value, where the terminal diameters were relatively close to the range of 210-600 μm (Fig. 3). Although *N*_2_ was set at 148 (Ii et al. 2020) because the pial arterial network was included in the fine arteries, we changed *N*_2_ to 37 to express reasonable diameter ranges of the fine arteries without the pial arterial network, as shown in Fig. 3. In the revised manuscript, we have noted the above descriptions on page 11, as follows.

“the case of (*N*_1_, *N*_2_) = (2000, 37) as a representative case, in which the terminal diameters of the coarse arteries were relatively close to the range of the intermediate anastomoses (210-600 μm (12))”

**Comment 11. In the paragraph starting on line 229, is the flow recovery ratio the same as the one described in lines 138-140 ? If so, the authors should refer to it for readability.**

*Response*

To clarify the definition of the flow recovery ratio, we have added the equation number for the definition of the flow recovery ratio on page 9, given by

$\text{Flow recovery ratio}=q_{\text{occlusion}}/q_{\text{normal}}$,

Furthermore, we have revised the explanation about the flow recovery ratio on page 11 to “the flow recovery ratio defined as eq. (4)” in the revised manuscript.

**Comment 12. Linking to the above comment, the authors show “spatial distributions of the flow recovery ratio”. How is this being computed ? if the same process as the one described in 138-140 is used, then this is a constant quantity in each subregion. I recommend including a more precise description.**

*Response*

The flow recovery ratio was computed in each subregion of the terminal of the pial arterial network (Fig. 3(c)), where the total number of terminals was 1,124,504 as noted on page 8 in the manuscript. In the revised manuscript, we have clarified the above on page 11, as follows.

“Note that the flow recovery ratio was computed in each subregion to which each terminal of the pial arteries was assigned, unless otherwise noted.”

**Comment 13. In Figure 3, include one or two sentences describing the observations from the plot to help the reader.**

*Response*

Following the reviewer’s suggestion, we have revised the caption of Fig. 3 with a further description of the observation, as follows.

“Fig. 3 Box plot of the terminal edge diameter of each coarse and fine arterial structure of three patterns at the number of coarse and fine arterial subregions (*N*_1_ and *N*_2_) = (1000, 74), (2000, 37), and (4000, 19). In all three patterns, diameter of the coarse arteries distributed in whole brain surface was in the range of *O*(10^2^) µm whereas that of the fine arteries locally distributed in the brain cortex was in the range of *O*(10^1^) µm.”

**Comment 14. In Figure 5, it shows a spatial distribution of flow on the surface of the brain. I think that a precise description of the way this is being computed should be included. The process mentioned in line 138-140 can be made more precise and can be referred to throughout.**

*Response*

As well as the response to comment 12, we have revised the description of the flow recovery ratio to clarify how it is computed in the caption of Fig. 5, as follows.

“The flow recovery ratio was computed in each subregion to which each terminal of the pial arteries was assigned, unless otherwise noted.”

**Reviewer #2**:

**Comment 1**. **Could the authors theoretically or mechanically explain why Tier 2 anastomoses have a higher impact on collateral blood supply? It is also interesting to compare effects of anastomoses between Tier 2 and Tier 1 (the circle of Willis). These investigations may help quantitatively understand relationships in a cerebral vascular network between the blood flow and vessel diameters and numbers at an anastomose. This may be also related to the flow recovery ratio in Fig. 7; a value at the infinite anastomosis number and a gradient may be related to geometries of vessel network.**

*Response*

We thank the reviewer for the interesting suggestions. The higher impact of the tier 2 anastomoses compared to tier 3 anastomoses can be qualitatively explained by higher pressure drops due to high hierarchical positions and lower hydraulic resistances due to larger diameters. These qualitative characteristics can be estimated theoretically using analogies to the electrical circuit, where anastomoses can be modeled as short circuits. However, these theoretical models need to treat the flow distribution as 0D systems, making it difficult to evaluate the effects of anastomoses on the spatial flow distributions, which are highly relevant to clinical observations. Therefore, we believe that the proposed multiscale anastomoses modeling is beneficial to deepen our understanding of the effects of multiscale anastomoses on the degree of collateral blood supply.

We agree with the reviewer’s suggestion that further comparison of the effects of tier 1 (Circle of Willis) and tier 2 anastomoses is interesting. Although tier 1 plays a minor role in the case of acute ischemic stroke caused by large vessel occlusion at the post-Circle of Willis, which is the target of this study, in cases where abnormalities occur at the pre-Circle of Willis, such as carotid artery stenosis, a full-scale anastomoses including tier 1 to 3 might help to preserve the cerebral blood flow distributions, as noted in our previous study (Yuhn et al. 2022). This study aims to develop a reasonable multiscale anastomosis in a cerebral arterial network, and a comprehensive understanding of a full-scale anastomosis is beyond the scope of this study. Nevertheless, further systematic analyses of the effects of multiscale anastomoses would deepen our understanding of the sustainability of the cerebral blood supply. In the revised manuscript, we have added the above further considerations on page 16, as follows.

“Fifth, this study investigated the effects of Tier 2 and Tier 3 anastomoses while the relative effects of Tier 1 anastomoses (Circle of Willis) were not considered because the Tier 1 anastomoses play a minor role in the case of acute ischemic stroke caused by large vessel occlusion at the post-Circle of Wills, which is the main target of this study. However, in cases where abnormalities occur at the pre-Circle of Wills, such as carotid artery stenosis, full-scale anastomoses including Tier 1 to 3 may help to ensure sustained blood supply, as noted in our previous study (11). Further systematic analyses of the effects of multiscale anastomoses in various abnormal conditions would provide a comprehensive understanding of the sustainability of the cerebral blood supply.”

**Comment 2**. **In L94, should “… (20) is provided …” be corrected to “… (20) are provided …” ?**

*Response*

Corrected.

**References in alphabetic order**

Ii, Satoshi, Hiroki Kitade, Shunichi Ishida, Yohsuke Imai, Yoshiyuki Watanabe, and Shigeo Wada. 2020. “Multiscale Modeling of Human Cerebrovasculature: A Hybrid Approach Using Image-Based Geometry and a Mathematical Algorithm.” *PLoS Computational Biology* 16 (6): e1007943.

Olufsen, M. S. 1999. “Structured Tree Outflow Condition for Blood Flow in Larger Systemic Arteries.” *The American Journal of Physiology* 276 (1): H257-68.

Vander Eecken, H. M., and R. D. Adams. 1953. “The Anatomy and Functional Significance of the Meningeal Arterial Anastomoses of the Human Brain.” *Journal of Neuropathology and Experimental Neurology* 12 (2): 132–57.

Yuhn, Changyoung, Marie Oshima, Yan Chen, Motoharu Hayakawa, and Shigeki Yamada. 2022. “Uncertainty Quantification in Cerebral Circulation Simulations Focusing on the Collateral Flow: Surrogate Model Approach with Machine Learning.” *PLoS Computational Biology* 18 (7): e1009996.
